# Deep-learning-based target screening and similarity search for the predicted inhibitors of the pathways in Parkinson's disease

**DOI:** 10.1039/c9ra01007f

**Published:** 2019-04-02

**Authors:** Abbas Khan, Aman Chandra Kaushik, Syed Shujait Ali, Nisar Ahmad, Dong-Qing Wei

**Affiliations:** School of Life Sciences and Biotechnology, Shanghai Jiao Tong University Shanghai 200240 China dqwei@sjtu.edu.cn; Center for Biotechnology and Microbiology, University of Swat Swat Pakistan

## Abstract

Herein, a two-step *de novo* approach was developed for the prediction of piperine targets and another prediction of similar (piperine) compounds from a small molecule library using a deep-learning method. Deep-learning and neural-network approaches were used for target prediction, similarity searches, and validation. The present approach was trained on records containing the data. The model attained an overall accuracy of around 87.5%, where the training and test set was kept as 70% and 30% (17 226/40 197), respectively. This method predicted two targets (MAO-A and MAO-B) and 101 compounds as piperine derivatives. MAO-A and MAO-B are important drug targets in Parkinson's disease. Validation of this method was also performed by considering piperine and its targets (monoamine oxidase A and B) using molecular docking, dynamics simulation and post-simulation analysis of all the selected compounds. Rasagiline, lazabemide, and selegiline were selected as controls, which are already FDA-approved drugs against these targets. Molecular docking studies of the FDA-approved drugs and the compounds we predicted using DL and neural networks were carried out against MAO-A and MAO-B. Using the molecular docking's scoring function, molecular dynamics simulation and free energy calculations as extended validation methods, it was observed that the compounds predicted herein possessed excellent inhibitory effects against the selected targets. Thus, deep learning may play a very effective role in predicting the potential compounds, their targets and can play an expanded role in computer-aided drug approaches.

## Abstract 2

Herein, a two-step *de novo* approach was developed for the prediction of piperine targets and another prediction of similar (piperine) compounds from a small molecule library using a deep-learning method.

## Graphical abstract

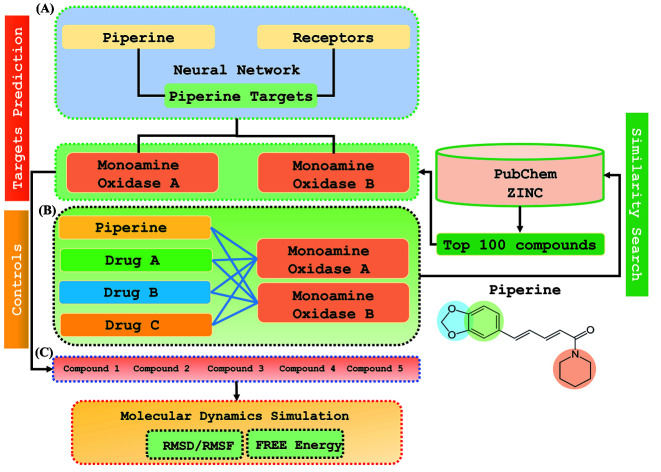

## Introduction

The remarkable growth of structure-based virtual screening techniques and confidence in these approaches have accelerated the drug-discovery process.^1,2^ These methods depend on the binding affinity scores between a target and a candidate molecule based on the 3D structure of their complex to predict the top hit molecules for onward processing and the following experimental investigation. The available scoring schemes are based on “statistical or expert” analysis of available protein–ligand structures.^3,4^ Various research studies conducted on drug discoveries witnessed the ever-increasing use of machine learning (ML) methodologies^5–7^ to identify the relationship in protein–ligand complexes. ML models convert these relationships into a scoring scheme (binding affinity scores), and these binding affinity scores provide a simple and better alternative to inferences based on the statistics and expert knowledge. In the ML approaches, the input data and expected results are provided to the model, and the model then predicts the outcome. A minute change in the model parameters can have incremental effects on the scores in an ML scoring system based on the RF-Score^8,9^ with an enabled random forest option and NNscore,^10,11^ primarily using neural networks. The outcome of this scoring system can be laudable in virtual screening to yield more active compounds as compared to the case of classical approaches.^9,12^ The growth of substantial structural data as well as affinity data provide a spur to researchers to explore them *via* deep learning approaches. In deep learning, the information contained in data is used to develop a meaningful relationship with the output. Therefore, the presentation of input data and its relationship with the output has a significant impact on the predictions of the model used.^13^ Continuous and prolonged research efforts in this field have enabled feature extraction to be developed in ML models. Thus in this approach, molecule representation is treated as the first part of the model. The molecule representation coupled with the predictive part is then used to extract features to solve specific tasks. This mechanism has been proved to be useful to find unknown and novel relationship.^14,15^

Deep learning is widely applied by bioinformaticians^16,17^ and computational biologists.^18^ In recent years, deep-learning methods have shown promising utilization in computer-aided drug design (CADD), where first structure-based approaches and then ligand-based models have been used. In the simplest deep-learning models, structure-based designs use molecular information as vectors to develop connected neural networks on top of them. These approaches return promising results to predict the bioactivity,^19^ aqueous solubility,^20^ and toxicity^21^ of the structures. Additionally, the multitask neural network model can predict the activities of multiple targets, and the results of the QSAR models are usually better than from single-task networks due to the better representation, training of data, and recognition of general patterns in the data.^7,19,21–23^ Neural networks are flexible and thus provide the best representation of data to the model, *e.g.*, by using convolution or a recurrent neural network to get patterns or an acyclic representation on the molecular graph.^24,25^

Numerous deep-learning studies have been conducted, where auto-encoders or recurrent neural network methods used in deep models have been used to propose new molecules with the desired properties.^26–29^ The application of deep-learning approaches to ligand-based and structure-based analysis has resulted in the development of various ligand-based and structure-based neural networks, such as AtomNet^30^ and other models like those proposed by ref. 31 and 32. In AtomNet, a molecular complex (input) is fed in to the convolutional neural network, which recognizes the interacting atoms and assigns a score of 1 for the active ligands and 0 for inactive ligands. The model proposed by ref. 31 was based on activity and prediction, while that in ref. 32 was based on the energy gap between a protein–ligand complex and the apo states. Deep learning utilizes a flexible architecture as compared to the other existing methods to design a problem-specific neural network (NN) to resolve it. Determining the protein–ligand interaction is the fundamental part of the molecular docking program, and for this, many scoring functions have been developed either on the basis of force fields or knowledge of existing complex protein–ligand structures.^33^

Considering the current findings and approaches above, we developed a two-step *de novo* approach using the PERL script, where specific inputs are used in order to have a good output efficiency. The overall dataset comprised a 70% training set and the remaining 30% was used as the test dataset. Piperine and eight targets were considered for training and testing the dataset for the prediction of piperine targets. Piperine and its compounds in PubChem and ZINC databases were used for training and testing the dataset for the prediction of similar compounds from small molecules libraries (ZINC and PubChem), with 101 compounds studied as potential inhibitors. The dataset was split in a 70/30 ratio for training and testing using the PERL script for our novel deep neural network tailored to 8 experimentally reported structures, and then for the derivative prioritization of piperine screening. The predicted top five compounds were then validated by comparing with experimentally reported FDA approved drugs (lazabemide, rasagiline, and selegiline) by using rational docking, molecular dynamics simulation, and free energy calculations. Overall these methods reported that the compounds predicted by our methodology possessed a higher potential than those of the drugs experimentally reported to be active.

## Materials and methods

### Deep learning approach

**Fig. 1:**
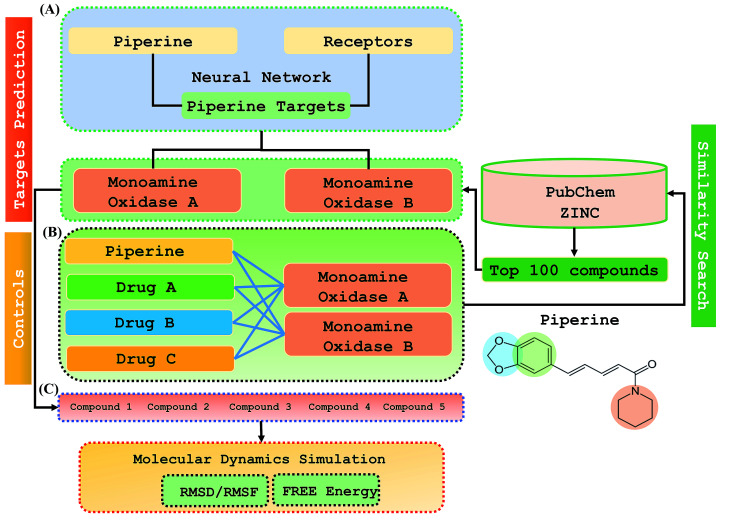
The overall flow of the work was divided into three parts: (A) prediction of the piperine targets based on a deep-learning methodology, (B) similarity search for prediction of the top 100 best hits based on piperine from the ZINC and PubChem databases using a machine learning approach, (C) validation and comparison of our predicted compounds with the FDA-approved drugs against the selected targets.

Step 1: A dataset was normalized using the PERL script, where specific inputs were used in order to achieve a good output efficiency. The dataset was divided into a 70% training set and the remaining 30% was used as the test dataset. The validation observed for the test dataset was about 87.5%. The overall flow of the work is given in Fig. 1.

Step 2: Piperine and eight targets were used for training and testing the dataset for the prediction of piperine targets. Piperine and 57 423 compounds were used for the training and testing of the dataset for the prediction of similar compounds from small molecules library (ZINC and PubChem), with 101 compounds studied as a potential inhibitors, with the dataset categorized in a 70/30 ratio into a training and testing set using the PERL script. These steps are given below:

Step1: Normalization of 57 423 compounds in the dataset.

Step2: Input the data for training:

(1) Prediction of the piperine targets' interrelated values of input and output to execute for training.

(2) Prediction of similar (piperine) compounds' interrelated values of input and output to execute for training.

Step 3: Set network constraint.

Step 4: Calculate the neurons of the output, with every neuron output signal calculated.

Step 5: Signal for the output layers calculation.

Step 6: Compute the error of the neuron and repeat step 3 and step 6 until the network is congregated and the error is computed.

RMSE and MAE (mean absolute error) were used to measure the prediction error. A correlation was assessed by using Pearson's correlation coefficient (*R*) and the standard regression (SD) deviation (i) methods.i
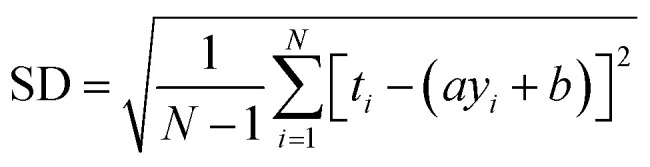
where *t*_*i*_ and *y*_*i*_ are the measured and predicted affinities for the *i*th complex, whereas *a* and *b* are the slope and the intercept of the regression line the between measured and predicted values, respectively.ii
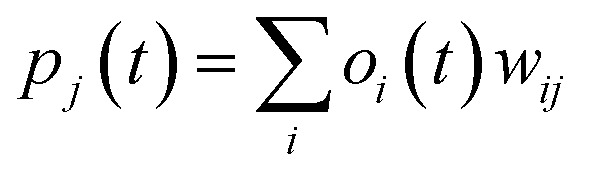

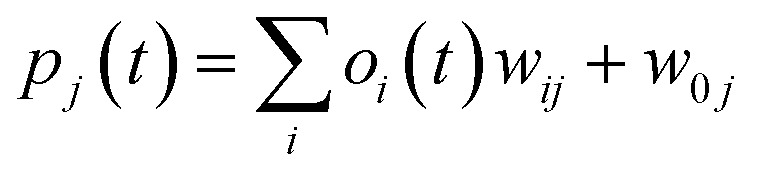
where *w*_0*j*_ is a bias.iii
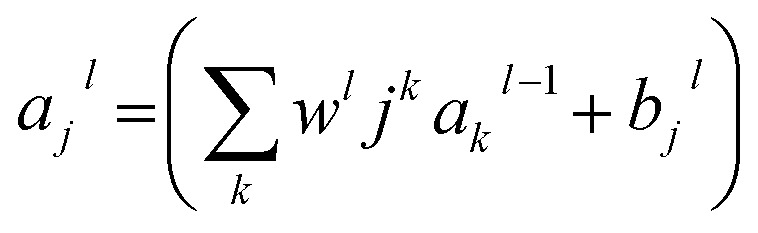
where the sum is over all the neurons *k*_*k*_ in the (*l* − 1)_th_(*l* − 1)_th_ layer. To rewrite this expression in a matrix form, we defined a weight matrix *w*_l_*w*_l_ for each layer, *ll*. The entries of the weight matrix *w*_l_*w*_l_ are just the weights connected to the *l*_th_*l*_th_ layer of the neurons, that is, the entry in the *j*_th_*j*_th_ row and the *k*_th_*k*_th_ column is *w*_*ljk*_*w*_*jkl*_. Similarly, for each layer *l*_*l*_, we defined a bias vector, *b*_*l*_*b*_*l*_. You can probably guess how this works—the components of the bias vector are just the values *b*_*lj*_*b*_*jl*_, *i.e.*, one component for each neuron in the *l*_th_*l*_th_ layer. Finally, we defined an activation vector *a*_*l*_*a*_*l*_ whose components are the activations *a*_*lj*_*a*_*jl*_.

### Validation

#### Molecular docking

The docking estimation was performed on the retrieved protein structures, namely monoamine oxidase A (PDB ID: 2BXS) and monoamine oxidase B (PDB ID: 1GOS), from the protein data bank (RCSB) (http://www.rcsb.org).^34^ MMFF force field was used to optimize the structure of piperine. An energy minimization step was performed using Powell's method and the default setting. The binding potential of piperine with protein was estimated by using the Lamarckian genetic algorithm in Autodock 4.0.^35^ The binding energies between the protein and ligand were estimated on a grid map generated by the AutoGrid program. The compounds with the lowest energy values were selected for onward processing.

#### Interaction pattern and poses analysis

The interactions of the selected ligands with MAO-A and MAO-B were sampled by using the Pymol Visualization tool^36^ and Protein–Ligand Interaction Profiler (PLIP) (https://projects.biotec.tu-dresden.de/plip-web/plip/index).^37^ The hydrogen bonding, electrostatic interactions, hydrophobic, and other interactions were visualized.

#### All atoms simulations

The AMBER 14 molecular dynamics package^38^ was used to conduct the MD simulations for all the selected complexes. The addition of Na^+^ ions and hydrogen helped to neutralize the systems counter with the application the “tleap” package in Amber. A TIP3P water box of 8.0 Å was used. Energy minimization of the complexes was carried out in AMBER 14 using the SANDER module at two stages (each of 6000 steps) in order to remove all the constraints atoms in the systems. PMEMD.cuda^39^ was used for the MD simulations. The SHAKE and Particle-Mesh Ewald (PME) methods with a non-bond contacts cutoff radius of 10 Å were used for the long-term interactions. Isotropic molecule-based scaling with 310 K (Langevin temperature) and (constant pressure) 1 atm was considered for 10 000 picoseconds equilibration time, followed by a total simulation of 20 ns. After every 2.0 ps time scale, MD trajectory sampling was performed. RMSD, RMSF, and hydrogen bonding were calculated by using CPPTRAJ and PYTRAJ.^40^ The following equation was solved to calculate the stability of the complexes after 100 ns.iv
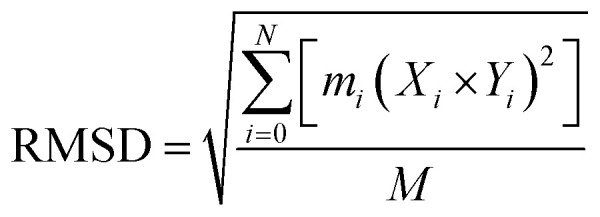
where, *N* = represents total atoms, *m*_*i*_ = mass of atom *i*, *X*_*i*_ and *Y*_*i*_ = coordinate vectors for the target and reference atom *i*, *M* = total mass.

#### Binding free energy calculation

The binding of ligands to MAO-A and MAO-B could be quantitatively measured by using MM-GBSA combined with MD simulation.^41^ For each molecular species, apo and holo, the *G*_bind_ (binding free energy) was calculated by using the following equation:

v (*G*_bind_) = *G*_R+L_ – (*G*_R_ + *G*_L_)

The different components (*G*_R+L_, *G*_R_, and *G*_L_) required for the free energy calculation of the apo and holo states are given in eqn (v). In the MM/GBSA and MM/PBSA methods, each free energy term in eqn (v) is calculated using the following equation:

vi *G* = *E*_bond_ + *E*_vdw_ + *E*_elec_ + *G*_PB_ + *G*_SA_ − *TS*_S_

In eqn (vi), *E*_bond_, *E*_vdw_, and *E*_elec_ are the bond energies, van der Waals, and electrostatic energy, including the dihedral bonds and angles, *G*_PB_ and *G*_SA_. *TS*_S_ represents the solvation energy corresponding to the polar and non-polar contributions, including absolute energy and solute entropy. The optimized parameters and MIEC model, as proposed recently, work for calculating the free energies between protein–protein interfaces,^42–45^ but here we utilized the MM-PBSA.py method using interior solute and exterior solvent values as constant^46^ to calculate the free energy.

## Results

### Deep-learning-based target screening and similarity search

**Fig. 2:**
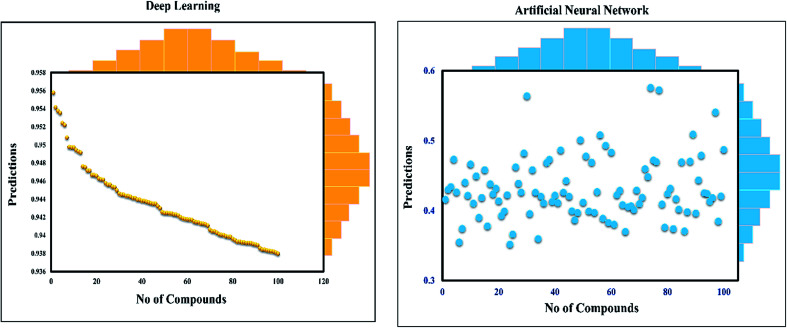
Prediction of neural connectivity where the graph represents the training, validation, testing, and target outputs. The graph shows the training, testing, and validation for drug screening, where a single curve indicates the comparative analysis of NN and deep-learning performance.

**Fig. 3:**
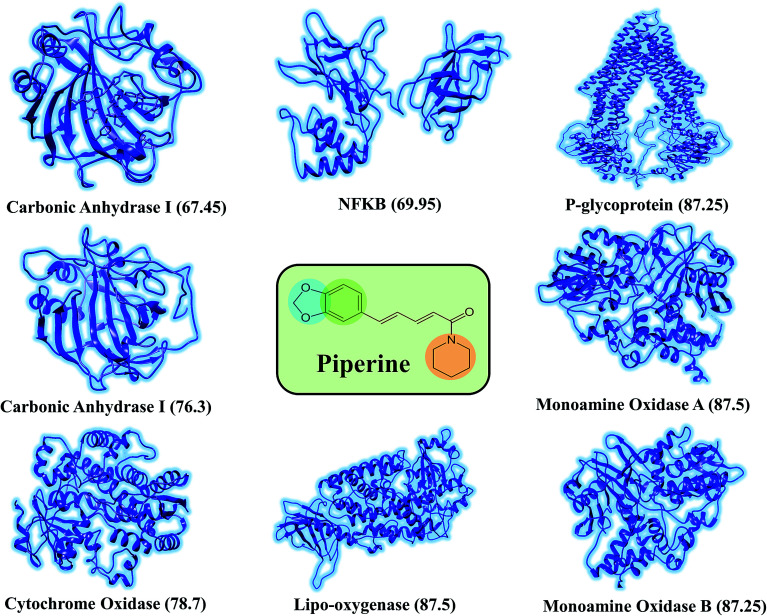
Depiction of the experimentally reported targets for piperine. The deep-learning-based scoring to predict the targets for piperine was applied to filter the top targets for piperine. Ranking of each target was carried out by the scoring given against each target.

This study was categorized into two parts. The first part predicted targets for piperine and potential compounds using piperine as the input. A deep-learning model was then used for the prediction of piperine's targets and its derivatives. The potential of the final predicted compounds was tested by using rational docking, molecular dynamics simulation, and free energy calculations. The pipeline was supplemented with FDA-approved drugs as controls. The approach was written in PERL script, where the prediction accuracy achieved was 87.5% based on a deep-learning network (Fig. 2). Piperine and eight targets (Fig. 3) were taken for training and testing of the dataset for the prediction of piperine targets; while Piperine and 57 423 compounds were taken for training and testing of the dataset for the prediction of similar compounds from small molecules library (ZINC and PubChem), where 101 compounds were studied as potential inhibitors.

**Table:** 

(1) Initialize the weight and parameters *μ* (*μ* = 0.01)
(2) Compute the sum of the squared errors overall input *F*_(*w*)_ = *eTe*, where the weight of network *w* = [*w*_1_, *w*_2_, *w*_3_,…*w*_*n*_) and ***e*** is the error vector for the network
(3) Solve to obtain the increment of weight *Δ*_*w*_ = [*J*^*T*^*J* + *μ*I]^−1^*J*^*T*^*e*, where *J* is a Jacobian matrix, *μ* is learning rate neither *μ* is multiplied by decay rate *β*_(0<*β*<1)_
(4) Using *w*+ *Δ*_*w*_
*F*_(*w*)_ < *F*_(*w*)_ then (go back to step 2)
*W* = *w*+ *Δ*_*w*_
*μ* = *μβ* (*β* = 0.1) (go back to step 2)
ELSE
*μ* = *μ*/*β* (go back to step 2)
END IF

### Validation of the predicted targets and compounds

#### Scaffold evaluation

**Fig. 4:**
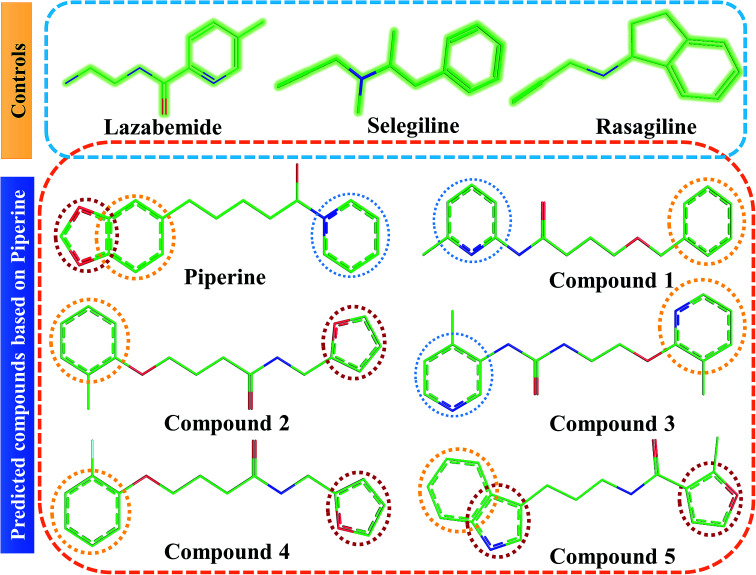
Chemical structures of the predicted compounds (panel B). Lazabemide, selegiline, and rasagiline in panel A were selected as controls to compare the activity of our predicted compounds.

The scaffold similarity of the predicted compounds and those of the FDA-approved drugs as control was carried out to mark the identity among these compounds. Fig. 4 shows the structural models of all the predicted and control compounds. It can be observed from the scaffolds that overall some rings that form hydrophobic interactions are similar to those of the control compounds. The backbones in the predicted compounds containing variable atoms significantly contribute to the formation of hydrogen bonding and thus produce strong inhibitory effects.

#### Ranking the interaction poses

Validation of our predicted best compounds against the selected targets was done by using the rational docking approach. To sample the best conformations of the predicted inhibitors in the active site, the 3D coordinates of the screened and selected targets (monoamine oxidase A and B) were retrieved from RCSB and prepared for docking simulation. Lowest energy conformational sampling, out of the total allowed five poses for each ligand, was done by analyzing the docking scores. For validation of our predicted compounds, we selected the top three active drugs against these targets as controls. The dataset containing the control and testing compounds was docked into the active pockets of our selected targets. The results showed that our selected compounds possessed stronger activity than the three control drug candidates. Table 1, summarizes the scores of each compound against the defined targets. Our induced-fit docking approach revealed that lazabemide, rasagiline, and selegiline possessed low binding affinities, specifically −6.06, −5.96, and −5.90 kcal mol^−1^, respectively, when compared to the predicted compounds. Among the deep-learning-based predicted compounds, compound 2 possessed the highest binding affinity −9.8 kcal mol^−1^ against MAO-A, followed by compound 3, with a binding affinity of −9.5 kcal mol^−1^, while compound 1 and 4 (−8.5 kcal mol^−1^) and compound 5 (−8.1 kcal mol^−1^) showed lower binding affinities but still better than the controls. These results suggest that our predicted compounds possessed better inhibitory property than those of the experimentally reported active compounds.

**Table 1:** Molecular docking based scoring of selected and predicted compounds against the selected targets monoamine oxidase A and B

S. no.	Compounds	Docking score	
		Monoamine oxidase A	Monoamine oxidase B
1	Piperine	−7.96	−7.7
2	Lazabemide	−6.06	−5.7
3	Rasagiline	−5.96	−6.1
4	Selegiline	−5.90	−6.3
5	Compound 1	−8.5	−9.6
6	Compound 2	−9.8	−9.3
7	Compound 3	−9.5	−8.8
8	Compound 4	−8.5	−9.5
9	Compound 5	−8.1	9.3

On the other hand, compounds such as lazabemide, rasagiline, and selegiline showed docking scores of −5.7, −6.1, and −6.3 kcal mol^−1^ against MAO-B. Docking of our deep-learning-based predicted compounds resulted in higher binding affinities compared to the control compounds. The total binding score for each compound (compound 1 to compound 5) was predicted to be −9.6, −9.3, −8.8, −9.5 and −9.3 kcal mol^−1^, respectively. These results suggest that our deep-learning-based method outperformed the controls and the predicted compounds had higher inhibiting potential than the experimentally active reported. It is also essential to explain that the predicted compounds possessed higher activity against MAO-B when compared to MAO-A.

**Fig. 5:**
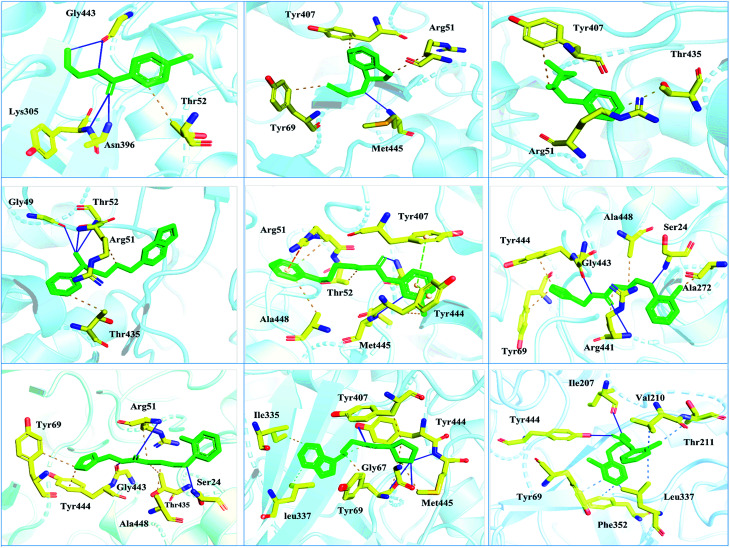
Interaction pattern of the selected FDA-approved drugs and the predicted top compounds with monoamine oxidase A. The ligands are colored in green, while the important interacting residues contributing hydrogen, hydrophobic, and electrostatic interactions are colored in yellow. Hydrogen bonding is colored in blue.

**Fig. 6:**
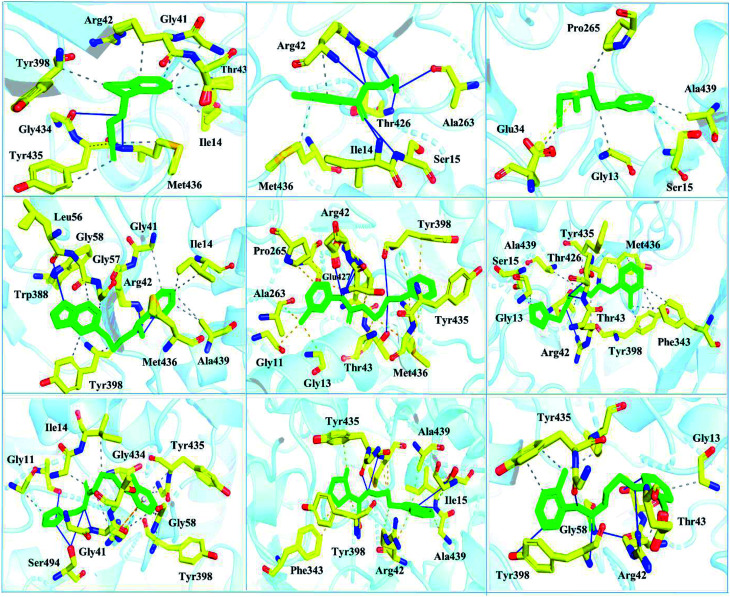
Interaction pattern of the selected FDA-approved drugs and the predicted top compounds with monoamine oxidase B. The ligands are colored in green, while the important interacting residues contributing hydrogen, hydrophobic, and electrostatic interactions are colored in yellow. Hydrogen bonding is colored in blue.

Interactions of the top ranking poses (Fig. 5 and 6) also showed that besides having the benzene ring in common responsible for hydrophobic interactions, we predicted the differences in the backbone of the compounds are strongly associated with the formation of hydrogen bonds with the active site residues. The docking scores of all the control and our predicted compounds are summarized in Table 1. These results confirmed that our methodology based on deep learning predicted potential compounds better than the already approved drugs and thus showed this technique could be applied to other targets for potential drug candidates discovery.

#### Stability analysis of the bound complexes

**Fig. 7:**
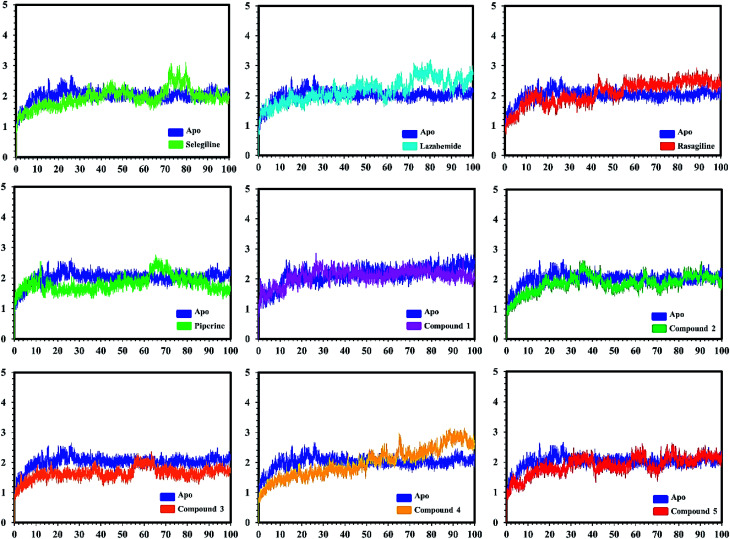
Conformational stability of the different complexes of monoamine oxidase A with the docked ligands during the MD simulations using Amber. The RMSD of the apo structure used as the reference structure is shown, with different colors showing the holo systems. The figure shows the number of frames on the *x*-axis, while RMSD (Å) is on the *y*-axis.

**Fig. 8:**
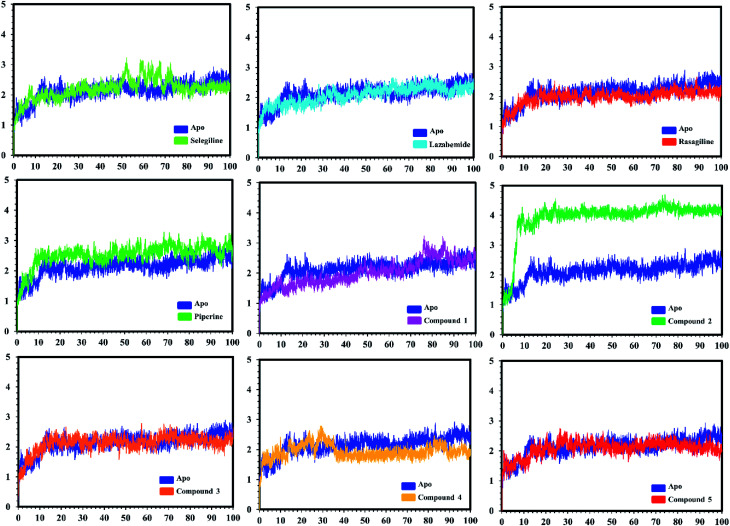
Conformational stability of the different complexes of monoamine oxidase B with the docked ligands during the MD simulations with Amber. The RMSD of the apo structure used as the reference structure is shown, with holo systems shown in different colours. The figure shows the number of frames on the *x*-axis, shows RMSD (Å) is on the *y*-axis.

Post-simulation analysis, such as root mean square deviation (RMSD), of all the selected complexes was carried out to test the stability of our predicted compounds in the active pockets. Both apo and holo systems were subjected to 100 ns simulation time. An initial analysis revealed that the average RMSD for all the systems lay between 1 Å and 2.5 Å, which confirmed the dynamic stability of all the systems. Acceptable fluctuations were observed in some systems, but later on, the production stage was stable until 100 ns. Fig. 7 and 8 show the RMSD graphs of all the systems. The complexes (protein–ligands) systems attained the equilibrium state in the first 10–20 ns. The RMSD increased up to 2.1 Å and then reduced to 1.5 Å. Afterward, the RMSD remained constant around 1.5 Å with acceptable flux. In the case of the selegiline-MAO-A complex, the system attained a weak equilibrium state around 2 Å in 70–80 ns as compared to the apo system. Lazabemide-MAO-A complex also lost its stability from 70 ns and onwards with little fluctuation. The compound 3-MAO-A system lost the equilibrium state from 52–60 ns and remained stable for the rest of the MD simulation. The RMSD analyses showed the stable behavior of the predicted ligand complexes to confirm the strong binding and thus inhibiting influence on the receptor.

In the case of monoamine oxidase B systems, for the selegiline-MAO-B complex, the system attained equilibrium soon after reaching 15 ns, but fluctuations up to 3 Å were also observed between 70–80 ns. In the case of compound 4-MAO-B, the complex showed higher fluctuations up to 4 Å until 8 ns, but later on, the system attained the equilibrium state and followed the stability path until the end. On the other hand, compound 4-MAO-B complex was unstable until 40 ns from the very beginning. Later on, the system remained stable for the rest of the simulation time. In the case of the other systems, little fluctuations in the acceptable range were observed, but overall the binding of ligands in the active site stabilized the systems by contributing a different bonding energy. These results suggest that the binding of our predicted compounds tightly occupied the binding sites of MAO-A and MAO-B and thus produced a strong inhibitory effect as compared to the other systems.

**Fig. 9:**
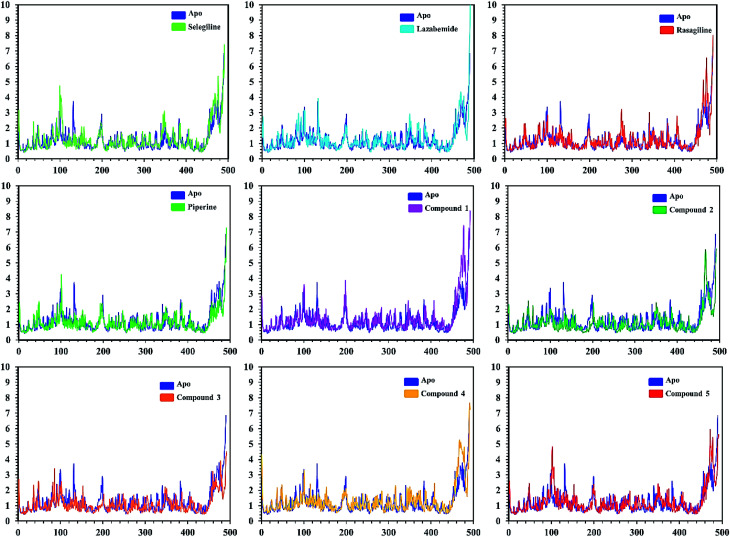
Cα RMSF of individual residues of all the apo and holo systems of monoamine oxidase A. Different colors show the RMSF of the apo structure and holo systems. The figure shows the number of residues on the *x*-axis, while RMSF (Å) is on the *y*-axis.

**Fig. 10:**
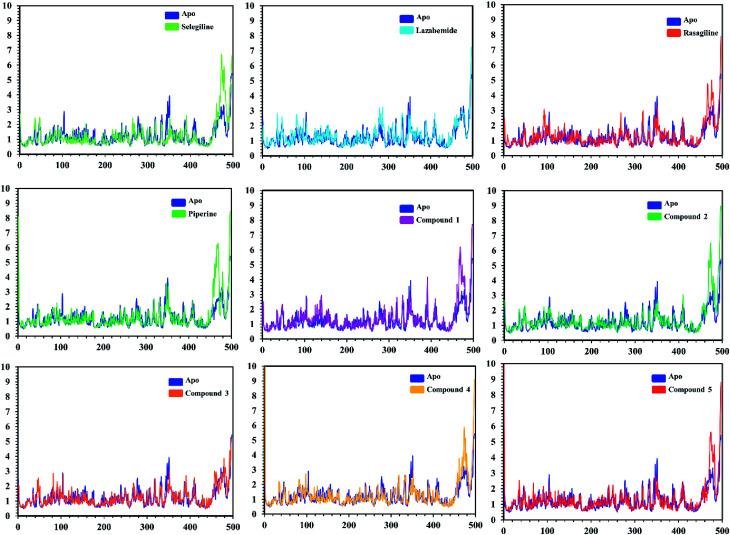
Cα RMSF of individual residues of all the apo and holo systems of monoamine oxidase B. Different colors show the RMSF of the apo structure and holo systems. The figure shows the number of residues on the *x*-axis, shows while RMSF (Å) is on the *y*-axis.

In order to find the residual fluctuations in MAO-A and MAO-B systems, both in the apo and complex state, root mean square fluctuation (RMSF) values of Cα were calculated. Fluctuations in both the apo and holo states of MAO-A were negligible. In all the systems, most of the fluctuations occurred in the N-terminus part. However, it was also observed that the binding of the inhibitor in the active site stabilized the systems by decreasing the residual fluctuation. In the case of MAO-B complexes when compared to the apo state, the C-terminus part also showed higher fluctuations. It is clear from the RMSF graphs (Fig. 9 and 10) that the binding of our predicted inhibitors significantly affected the residual fluctuation of the complexes.

#### Binding free energy analysis

**Fig. 11:**
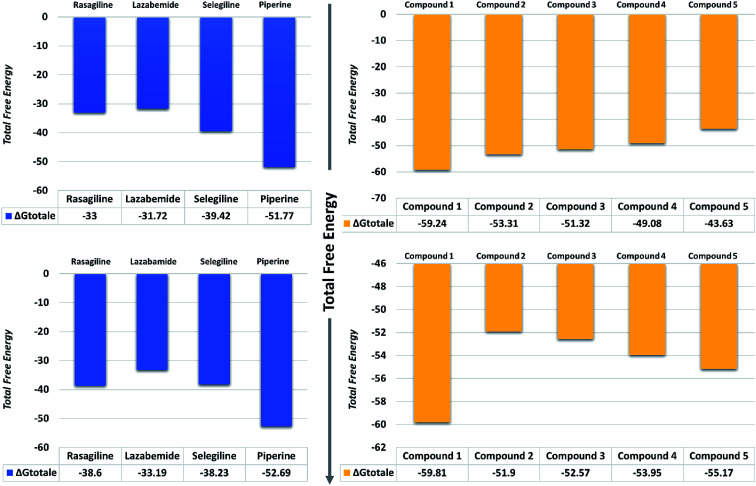
Total free energy of the controls and predicted compounds against monoamine oxidase A and B are given in the graphs. All the energies are calculated in kcal mol^−1^.

MM/PBSA and MM/GBSA methods are popular approaches to estimate the free energy of the binding of small ligands to biological macromolecules. In order to validate the accuracy of our method, the predicted top 5 ligands and also the controls were subjected to free energy calculations (Fig. 11). The results from the calculations on MAO-A-ligands and MAO-B ligands complexes suggested that our predicted compounds were stronger inhibitors than those already reported. Energies calculations on MAO-A-ligands complexes reported that the predicted compounds were sequentially stronger inhibitors than the controls. Considering the total free energy (Δ*G*_bind_) as tabulated in Table 2, it is suggested that compound 1 possessed stronger binding affinity (−59.24 kcal mol^−1^) energy, followed by compound 2 (−53.31 kcal mol^−1^), compound 3 (−51.32 kcal mol^−1^), compound 4 (−49.08 kcal mol^−1^), and finally compound 5 (−43.63 kcal mol^−1^), respectively.

**Table 2:** Binding free energies of the predicted inhibitors to monoamine oxidase A and monoamine oxidase B calculated by using MM-GBSA approach. Rasagiline, lazabemide, selegiline, and piperine were grouped as the controls. Compounds 1–5 are our predicted compounds based on the similarity search

Targets	Experimental compounds	*Δ*_vdW_ a	*Δ*_ele_ b	*Δ*_SASA_ c	Δ*G*_bind_^d^
Monoamine oxidase A	Rasagiline	−34.36	−0.29	−3.34	−33.00
	Lazabemide	−33.69	−0.22	−3.10	−31.72
	Selegiline	−40.77	−0.54	−4.13	−39.42
	Piperine	−56.88	−4.15	−5.49	−51.77
	Compound 1	−64.16	−3.15	−6.07	−59.24
	Compound 2	−57.56	−0.85	−5.53	−53.31
	Compound 3	−59.44	−4.83	−5.52	−51.32
	Compound 4	−52.80	−2.95	−5.49	−49.08
	Compound 5	−45.60	−2.29	−5.51	−43.63
Monoamine oxidase B	Rasagiline	−40.56	−1.71	−3.56	−38.60
	Lazabemide	−35.01	−0.27	−3.44	−33.19
	Selegiline	−40.44	−0.35	−4.28	−38.23
	Piperine	−57.66	−4.19	−5.25	−52.69
	Compound 1	−63.52	−3.26	−5.91	−59.81
	Compound 2	−57.01	−2.02	−5.61	−51.90
	Compound 3	−58.30	−4.59	−5.79	−52.57
	Compound 4	−61.57	−4.22	−5.30	−53.95
	Compound 5	−60.75	−3.984	−5.444	−55.17

a van der Waals energy.

b Electrostatic energy.

c Solvent accessible surface area energy.

d Total binding free energy.

On the other hand, the compounds taken here as the controls produced weaker binding energies as compared to our predicted compounds. Specifically, the total free energies for rasagiline, lazabemide, and selegiline were reported to be −33.00, −31.72, and −39.42 kcal mol^−1^, respectively. The total free energy for piperine, which was considered as an input for the similarity search, was reported to be −51.77 kcal mol^−1^, which is ultimately better than the three selected controls. It could be inferred from these binding energies against MAO-A that our predicted compounds could efficiently inhibit MAO-A better than those of the already experimentally reported compounds.

The binding affinities of ligands and MAO-B were also calculated from the last 10 ns of the MD trajectory. As can be seen from Table 2, the total Δ*G*_bind_ values of rasagiline, lazabemide, and selegiline were −38.60, −33.19, and −38.23 kcal mol^−1^, respectively. The results of our top predicted compounds showed values of −59.81, −51.90, −52.57, −53.95, and −55.17 kcal mol^−1^, respectively, which confirmed the strong inhibition properties of these compounds. The interaction of piperine with MAO-B also possessed strong binding affinity by contributing a total energy of −52.69 kcal mol^−1^. These results essentially validate our prediction method and thus the reported novel ligands that could robustly inhibit these targets.

## Discussion

The discovery of novel small molecules with strong inhibitory potential is a common practice used by researchers. Essential drug features, such as HBD, HBA, and others, are used by computational chemists to find novel drug candidates based on these defined features. Machine-learning methods, such as ANN, have long been used in the prediction of molecule activity. Generally, DL strategies are enacted in the first place to handle the issues of activity prediction. When compounds are portrayed with the same number of molecular descriptors, researchers use fully connected DNNs to build models, which is considered a straightforward method.^47^ Evaluating the interaction between a protein and a ligand is the key element in the molecular docking program, and many scoring functions have been built up either by force fields or using the knowledge of existing complex protein–ligand structures to assist this process.^48^ A typical example is given in the investigation done by Ragoza *et al.*^31^

In this work, a deep neural network combined with a ML approach was used as a scoring function in the virtual screening or as an affinity predictor for novel molecules after a complex is generated. It can be either applied to test multiple compounds against a single protein or to test multiple proteins against a single compound. The model was applied to a single drug, namely piperine, and its experimental targets. A general docking approach and molecular dynamics simulation approaches were used as supplementary validation methods to investigate the potential of the predicted compounds against the prioritized targets. A total of eight experimental targets were selected, including TRPV1,^49^ nuclear factor-κB,^50^ monoamine oxidase A, monoamine oxidase B,^51^ carbonic anhydrase I, carbonic anhydrase II,^52^ lipoxygenase,^53^ P-glycoprotein I and CYP3A4,^54^ which were reported to be inhibited by piperine. Our deep-learning-based approach discovered that piperine could efficiently inhibit MAO-A and MAO-B. Monoamine oxidase (MAO) catalyzes primary, secondary, and tertiary amines and is considered one of the essential enzymes in neurotransmitter metabolism. Its physiological roles and inhibitors play a significant role in understanding the functional roles of dopamine (DA), norepinephrine, and serotonin (5-HT) neurotransmission in the central nervous system (CNS). It is, therefore, an essential drug target for the treatment of Parkinson's disease.

FDA-approved experimental ligands, such as rasagiline, lazabemide, and selegiline, were compared to piperine by using a conventional docking approach, which reported that piperine is better than all the others. Using a machine-learning approach, piperine was considered as an input for similarity search considering its inhibitory features. PubChem and ZINC databases were subjected to similarity searches to obtain the top 100 hits. Using ML scoring function, only the top 5 compounds were selected for further evaluation to evaluate the prediction power and accuracy of our method. Molecular docking, molecular dynamics simulations, post-simulation analyses, and free energy calculations confirmed that the compounds we predicted based on piperine were more potent inhibitors of MAO-A and MAO-B. Interaction pattern evaluation helped in understanding the bonding pattern. It was observed that the extra ring in the predicted compounds structures and different atoms in the backbones potentially formed hydrogen bonds with the active site residues. Overall the performance of our prediction method outperformed the controls by predicting the most potent compounds. It can, therefore, help in discovering new potential drugs, but also in investigating the side effects of bioactive molecules. By anticipating the potential impact of new drugs on the biology of the cell, deep-learning approaches may contribute to such disciplines as systems medicine and systems biology. The results obtained and the careful analysis of the results revealed reliable predictions based on relevant features. Thus, deep learning and ML-based features can significantly increase the reliability and accuracy of predicting novel inhibitors.

## Conclusion

This study was based on deep learning and machine-learning approaches to determine the impact of these state-of-the-art methods in predicting novel compounds against disease-causing targets. The prediction of targets and then similarity searches predicted potential compounds based on already approved drugs. Integrated MD simulations and free energy calculations revealed that the predicted compounds possessed stronger inhibitory potential than those of the already FDA-approved compounds, thus showing the enhanced reliability and accuracy of our method.

## Author's contribution

AK and ACK did all the analysis. DQW, SSA and NA formulated the method and wrote the manuscript. DQW is an academic supervisor who revised the manuscript. All the authors approved the manuscript.

## Conflicts of interest

The authors declare that there are no competing interests.

## Supplementary Material
